# A Systematic Review of Interventions that Use Multidisciplinary Team Meetings to Manage Multimorbidity in Primary Care

**DOI:** 10.5334/ijic.6473

**Published:** 2022-10-18

**Authors:** Elena Lammila-Escalera, Geva Greenfield, Susan Barber, Dasha Nicholls, Azeem Majeed, Benedict W. J. Hayhoe

**Affiliations:** 1 Imperial College London, UK; 2 Chelsea & Westminster Hospital NHS Foundation Trust, UK

**Keywords:** integrated care, primary care, multidisciplinary teams, multimorbidity

## Abstract

**Introduction:**

Multidisciplinary team (MDT) meetings could facilitate coordination of care for individuals living with multimorbidity, yet there is limited evidence on their effectiveness. We hence explored the common characteristics of MDT meetings in primary care and assessed the effectiveness of interventions that include such meetings, designed to improve outcomes for adults living with multimorbidity.

**Methods:**

A systematic review of literature was conducted using MEDLINE and EMBASE. A narrative synthesis was performed, extracting study and MDT meeting characteristics, in addition to any outcomes reported.

**Results:**

Four randomised controlled trials that were conducted in the United States of America were identified as eligible, recruiting a total of 3,509 adults living with multimorbidity. Common MDT meeting themes include regular frequency of discussion, the absence of patient involvement and the participation of three or four multiprofessionals. Significant improvements were observed in response to interventions with an MDT component across most measures, yet this trend did not extend to physical health outcomes.

**Discussion:**

It is unclear if the results in this review are sufficient to support the widespread implementation of MDT meetings in primary care, for adults living with multimorbidity. Due to the paucity of studies collated, further research is required to inform widespread implementation.

## Introduction

As life expectancies increase, a greater proportion of our population is vulnerable multimorbidity [1]. The prevalence of multimorbidity in individuals presenting to primary care ranges between 13.0% and 82.6%, dependant on the patient population, age parameters and the definition of multimorbidity applied [2,3,4]. Thus, multimorbidity is one of the largest challenges facing health systems today, contributing to a significant economic burden and around 70% of national healthcare expenditure in the United Kingdom [5].

Traditional health systems are poorly adapted to deliver care for people with multimorbidity. Disease-centric models of clinical management address each condition separately, delivering care for one disease at a time. This can result in inappropriate clinical attention and overtreatment [6], neglecting psychosocial status, preferences, and health goals. To improve the quality of care for individuals with multimorbidity, there has been a shift towards the provision of integrated care [7]. Integrated care centralises actions across multiple specialties and can improve both quality of care and life [8].

One core aspect of integrated care is interprofessional collaboration in the diagnosis, management, and treatment of multimorbidity [9]. Interprofessional care focuses on the importance of collaboration between multiple care providers, aiming to improve continuity of care, which has been linked to better health outcomes [10]. Effective collaboration between primary care physicians and mental health specialists also improves outcomes for patients with comorbid medical and psychiatric complications [11]. However, barriers to collaboration can include insufficient and delayed communication, due to service fragmentation [12].

Multidisciplinary teams (MDTs) are an important component of integrated care delivery [13]. MDTs are defined as a group of professionals, that can include nursing, medical and allied healthcare professionals, working together to improve outcomes for patients [14]. This enables the crossing of professional boundaries to ensure collaborative care. Cross-sector partnerships may maximise the contributions of service providers, promote comprehensive care coordination and improve quality of care [13,15].

MDT meetings are often recommended as a critical aspect of integrated care in guidance and opinion pieces, yet it is not clear how and to what extent this approach improves outcomes for patients with multimorbidity. Previous systematic reviews either focus on specific comorbidities, explore the efficacy of asynchronous collaboration, or synthesise evidence of MDT management in secondary care settings [16]. In 2012, Smith *et al* performed a systematic review of a broad range of interventions for multimorbid patients in primary care but did not focus on the characteristics of MDT meetings, nor reported their efficacy [17]. In the absence of evidence to inform a clear clinical consensus among decision-makers, the wide-spread implementation of MDTs in primary care might be left underutilised and remain a lost opportunity to improve patient care, regardless of the potential benefits.

This review aims to fill this knowledge gap by assessing the common characteristics and the effectiveness of interventions that include MDT meetings based in primary care, designed to improve outcomes for adults living with multimorbidity.

## Methods

A systematic review was conducted following the recommendations in the Cochrane Collaboration Handbook and ‘Preferred Reporting Items for Systematic Reviews and Meta-Analysis’ (PRISMA) Guidelines **(Appendix A)**.

### Eligibility criteria

This review employed the ‘Population, Interventions, Comparators, Outcomes, Study design approach’ at screening, for study eligibility [18].

#### Population criteria

The population of interest were adults diagnosed with multimorbidity or comorbidity, presenting to primary care. The WHO definition of adults was applied, namely individuals aged 19 years or older [19]. Multimorbidity was defined as the coexistence of two or more concurrent chronic conditions in an individual [20]. Although multimorbidity is a new MESH heading, this term was historically used synonymously with ‘comorbid’, so we included this and other historical synonyms. This review did not exclude by chronic disease.

#### Intervention criteria

Eligible studies focused on assessing an intervention including multidisciplinary collaboration in primary care described as (I) a meeting or discussion, (II) synchronous (same place/same time), (III) regarding a multimorbid patient case/s and (IV) either in the presence or absence of, that patient with multimorbidity.

The WHO definition of ‘multiprofessional’, a synonym for multidisciplinary, was used: “when three or more professions learn or practice together to improve health outcomes” [21]. Alternative synonyms for collaboration and primary care were also included.

#### Comparator criteria

The comparator was either the usual or standard care given to patients who attend primary care and present with multimorbidity.

#### Outcome measures

The heterogenous nature of multimorbidity suggested that a wide variety of potential outcomes may be recorded by relevant studies. After examining previous literature, we expected that physical health, mental health, functional health outcomes would be reported, in addition to frequency of utilisation of health services, patient behaviour, provider behaviour, acceptability, patient satisfaction with service provided and cost-effectiveness.

#### Study design criteria

We considered studies with experimental and observational designs, providing quantitative data. Qualitative designs and case reports were excluded.

### Search strategy

#### Information sources

Studies were identified by searching electronic databases, scanning reference lists of articles and previous systematic reviews. No limits were applied for language. This search was applied to MEDLINE and EMBASE, (inception to present). The search time frame was selected to encompass all possible interventions that include MDT meetings for individuals with multimorbidity. The search strategy, developed with a Specialist Librarian experienced in evidence synthesis, incorporated all relevant MESH terms, and is detailed in **Appendix B**.

### Selection process

Eligible studies were deduplicated and screened independently by two reviewers (ELE and SB). Discrepancies were resolved by discussion with the other authors.

### Data collection process

Data relevant to the research question were extracted and collated. Data extracted included the full description of the MDT meeting. The extraction table was based on the 10 key features of an ideal interdisciplinary meeting for patients with multimorbidity in primary care, identified by Delphi panel methodology [22].

### Quality assessment

This assessment was guided by Cochrane ‘Randomised Controlled Trial Risk of Bias 2’ (RoB2) tool and assessed independently by two reviewers (ELE and SB).

### Data analysis

Due to heterogeneity amongst the included interventions, a meta-analysis was not possible. Instead, a descriptive narrative synthesis was used to identify common MDT themes and outcomes from the extracted data.

## Results

### Study selection

**Figure 1:**
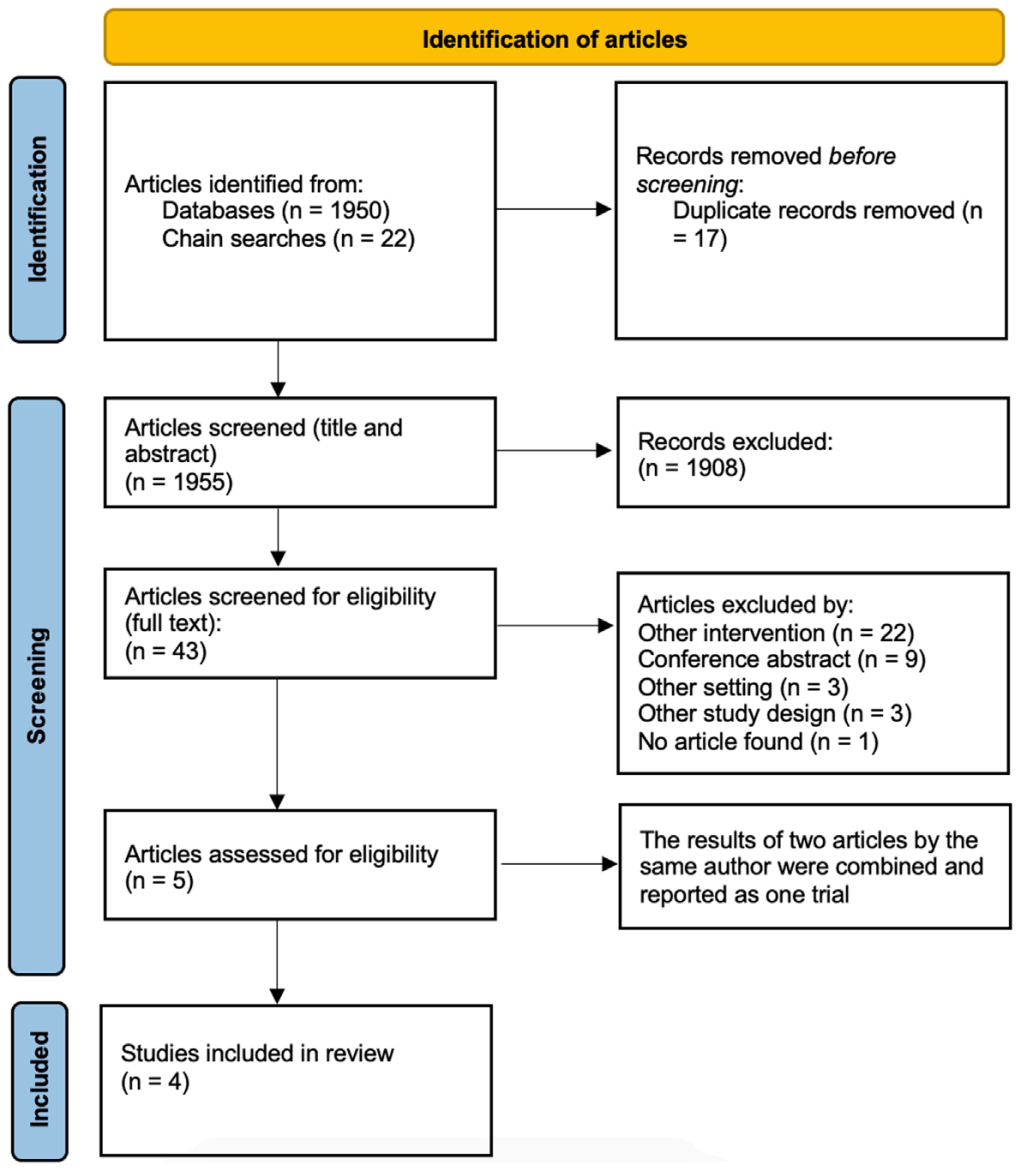
**The search results**. PRISMA 2020 Flow Diagram detailing the identification of relevant articles. Abbreviations: n; number.

An outline of the selection process is detailed in Figure 1. 1950 potentially eligible studies were identified through the database searches. A further 22 studies were identified by chain-searching reference lists of relevant studies, but most were excluded, being conference abstracts. Of the 43 studies selected for full-text screening, 28 were excluded as they did not meet the inclusion criteria in terms of intervention, setting, or study design. Five studies were initially identified, but the results of two studies were combined and are reported as one trial [23,24]. Four RCTs were therefore included in this review.

### Study characteristics

The four studies (Table 1) [24,25,26,27] were conducted between 2000 and 2012, all in the United States (USA) and recruited a total of 3509 participants. The trials varied in duration and follow-up, from 12 months to 24 months. One out of the four trials included patients with a broad variation of chronic disease [27], while the others focused on specific comorbidities [24,25,26].

**Table 1:** Study Characteristics.


TRIAL	DURATION AND FOLLOW-UP	STUDY PARTICIPANTS	SETTING	CONTROL	INTERVENTION	MDT MEETING DESCRIPTION	OUTCOMES	RESULTS

**Counsell et al 2007** [25]	Intervention: 24 months Follow-up: 6, 12, 18 and 24 months	Aged ≥ 65, Annual income 200% < federal poverty level, comorbidities (n = 951)	Primary care practice serving approximately 6000 patients	Usual care (n = 477)	Geriatric care management model: GRACE intervention (n = 474)	Weekly interdisciplinary team meetings (nurse practioner, social worker, primary care physician) to review support team success in implementing care protocols and problem solve barriers to implementation	Physical health, Function health, Utilisation of health services,	Improved scores in 4/8 components of the SF-36 in intervention group participants compared to standard care No differences observed in ADL scores between intervention group and standard care Mortality rate is reduced in the intervention group in comparison to standard care Hospitalisation and ED visits were lower in intervention group participants in the last 12 months of the trial,

**Harpole et al 2005** [26]	Intervention: 12 months Follow-up: 3, 6, 12 months	Aged ≥ 60, Major depression or dysthymia and ≥ 1 other chronic condition. (n = 1801)	18 primary care clinics	Usual care (n = 895)	IMPACT intervention (n = 906)	The district care nurse met weekly with the supervising psychiatrist and the liaison primary care physician to monitor progress and adjust treatment plans as needed	Mental health, Functional health	Significantly lowerSCL-30 depression scores in intervention patients compared to usualcare Improved MCS-12 scores at the 3- and 12- month interval in intervention group participants, in comparison to standardcare Improved scores of quality of life in the interventiongroup compared to those that received standard care

**Katon et al 2010/2012** [23,24]	Intervention: 24 months Follow-up: 6, 12, 18, 24 months	Depression and diabetes, or coronary heart disease, or both (n = 214)	14 primary care clinics	Enhanced usual care (n = 108)	TEAMcare program (n = 106)	Nurses met weekly for systematic case reviews with the family physician, consulting psychiatrist and internist, to enhance care coordination and ensure accountability for follow-up to guideline level disease management and achieve clinical goals	Physical health, Mental health, Functional health, Provider behaviour, Acceptability of services, Costs and cost-effectiveness	Improved LDL cholesterol levels, systolic bloodpressure 12 months, but the intervention group displayed no differencesat the 18- and 24 months interval Improved SCL-20scores within intervention participants The intervention group participants had 114additional depression-free days and an additional 0.335 QALYS Morelikely to have drug adjustments Intervention group participants experienced agreater satisfaction with their care in comparison to patients whoreceived standard care The intervention was cost-effective

**Sommers et al 2000** [27]	Intervention: 24 months Follow-up: 12 months post-intervention	Aged > 65, ≥ 2 chronic conditions (n = 543)	18 primary care clinics	Usual care (n = 263)	Collaborative Care (n = 280)	The physician, the nurse and the social worker met at least monthly to review each patient’s status and revise care plans.	Physical health, Functional health, Utilisation of health services, Costs and cost-effectiveness	Improved hospitalisation rate, mean primary care physician officevisits among the intervention group participants in comparison to thosewho received usual care Increase in social activity The interventionwas cost-effective


Abbreviations: SF-36; short form survey 36, MDT; multidisciplinary team, ADL; activities of daily living, ED; emergency department, SCL-30; check list of symptoms 30, GRACE; geriatric resources for the assessment and care of elders, IMPACT; improving mood-promoting access to collaborative treatment, LDL; low-density lipoprotein, n; number of participants.

### Description of the multidisciplinary team meetings

All the trials examined interventions that included MDT meetings (Tables 1 and 2). Three reported the frequency of meetings as weekly [24,25,26], whereas the other reported monthly meetings [27]. One study reported more than three healthcare professionals with differing disciplines [24], whereas the remaining studies reported three participants [25,26,27]. There were shared themes of surveillance, review, and goal setting. Factors such as the definition, duration, number of patient cases to address per meeting, structure and dissemination were not reported. No study attempted to correlate outcomes with specific intervention components.

**Table 2:** Reporting of the 10 key features of multidisciplinary team meetings in primary care, when managing the care of individuals living with multimorbidity.


FACTOR	DESCRIPTION	APPEARS IN STUDIES

**Definition**	Periodic gathering of different professionals who provide care for the multimorbid patients; for transdisciplinary discussion and adoption of clinical/and or organisational decisions	NR

**Ideal setting**	Meeting room (or another room with appropriate conditions)	NR

**Duration**	Less than 60 minutes, not exceeding 120 minutes in length	NR

**Frequency**	Every 2 weeks – dependant on number and complexity of multimorbid patients. Should not exceed a one-month interval	[23,24,25,26,27]

**Number of participants**	All the necessary players, considering the capacity of the room	[23,24,25,26,27]

**Professional presence**	Family physicians should always be present. Other health professionals should also be present: hospital doctors, nurses, social worker, psychologist, physiotherapist, pharmacist, and nutritionist.	[23,24,25,26,27]

**Patient presence**	Normally not, except if necessary to expose the clinical case or if the estimated treatment burden imposes the need for the patient’s presence to decide therapeutic options	[23,24,25,26,27]

**Number of patient cases to address per meeting**	Due to the complexity of the multimorbid patient, approach up to two clinical cases per meeting. This number will vary depending on the team’s experience in dealing with multimorbidity and the frequency and the duration of the meetings.	NR

**Structure**	A chairman of these meetings should be appointed to identify, leading the meeting. Each case should be presented by the family doctor or nurse, listing difficulties/doubts in management, followed by a discussion and a final definition of the consensus interventions. A facilitator is assigned.	NR

**Dissemination**	The results of the meeting regarding the management of the patients should be shared with all care providers in an effective and tailored way for each health professional, the patient, or their caregiver.	NR


The common themes between the studies reported regarding the multidisciplinary team meetings that took place within the intervention, sorted by the factors that contribute to an efficient multidisciplinary team meeting for patients with multimorbidity, based in primary care [22]. Abbreviations: NR; not reported.

### Bias

Overall, the studies had a low risk of bias. Randomisation, the blinding of the outcome assessment and the reporting of outcomes were comprehensive; allocation concealment, blinding of participants and healthcare professionals were less reliably reported.

### Outcomes

#### Physical health outcomes

Katon *et al* initially reported statistically significant improvements in systolic blood pressure glycated, haemoglobin and low-density lipoprotein levels in the intervention group in comparison to the control group [23,24]. However, these improvements were not sustained long-term, as differences in the intervention diminished over time [23,24]. Similarly, Counsell *et al* and Sommers *et al* observed no significant improvement in mortality rate among intervention group participants in comparison to those that received usual care, after 24 months [25,27].

#### Mental health outcomes

Harpole *et al* reported statistically significant reduction in Symptom Checklist (SCL-20) and Mental Health Component Scale (MSC-12) scores across all follow-up time points [26]. Katon *et al* concluded with a similar assessment, recording a significant reduction in SCL-20 depression scores for participants involved in the intervention, which was sustained throughout the trial duration. Furthermore, their intervention participants experienced 114 additional depression-free days in comparison to the control group [24].

#### Functional health outcomes

Counsell *et al* observed a significant improvement after 24 months in four Short Form Health Survey (SF-36) scale scores: general health, vitality, social functioning and mental health [25]. The authors further reported an ‘Activities of Daily Living’ score, on which no statistically significant difference was found [25]. Sommers *et al* revealed that for their ‘Social Activities Count’ outcome there was a significant improvement [27].

In addition, both Harpole *et al* and Sommers *et al* reported an improved quality of life [26,27]. Katon *et al* detailed that their intervention patients had an additional 0.335 QALYS in comparison to the group receiving standard care [24].

#### Utilisation of health services

Counsell *et al* reported no significant differences between the intervention and the usual care group regarding the emergency department attendance rate and hospital admission rate [25]. However, at 24-months, the cumulative emergency department attendance rate was significantly lower in the intervention group [25]. Sommers *et al* also observed that the rate of hospitalisation did not improve, instead remaining at baseline. However, it was reported that hospital readmissions and primary care physician office visits were significantly reduced [27].

#### Provider behaviour

Katon *et al* reported pharmaceutical measures relating to prescription behaviour and medication management, by the provider involved [23,24]. The intervention group was significantly more likely to experience medication reviews of insulin, antihypertensive medication, and antidepressant medication, compared to the group that received standard care [23,24].

#### Satisfaction with services

Katon *et al* reported a significantly greater satisfaction of care for diabetes, coronary heart disease or both, in addition to the care received for depression provided throughout the intervention, in comparison to standard care [23,24].

#### Costs and cost-effectiveness

Sommers *et al* estimated per-patient savings $90 associated with providing care to the intervention group [27]. Katon *et al* performed a cost-effectiveness analysis [24], and found that the cost per patient for the intervention was estimated to be $1224 and cost-effective. The incremental cost-effectiveness ratio found mean cost savings of $1773 per quality-adjusted life-year and under the $20,000 NICE willingness-to-pay threshold, reducing outpatient costs by $594 [24,28].

## Discussion

### Main findings

It is uncertain whether the evidence generated by this review supports the implementation of MDT meetings in primary care settings for individuals with multimorbidity. However, despite the limited number of studies, the variation in both participants characteristics and interventions were substantial. Additionally, the complexity of interventions meant that causality on any subsequent outcomes could not be attributed to the MDT meeting alone.

Although similarities were identified when exploring the characteristics of the MDT meetings, the reported quality of the MDT meetings was poor. There was an absence of detail regarding meeting duration, structure, and dissemination. The optimal frequency of MDT discussion is twice a month, to provide opportunity to review patient cases [22]. The results of this review support this statement, with studies reporting meetings of regular frequency in throughout the duration of the interventions. Participation involved either three or four multiprofessionals, with disciplines varying from primary care physicians, social workers, psychologists, and nurses. No element of patient involvement or co-creation of care was integrated within the MDT meetings. Although co-production is not a mandatory recommendation, it is beneficial to include patients in discussions about their care to ultimately improve outcomes [29,30]. Due to a lack of detail, it is difficult to draw generalisable conclusions regarding the efficacy of these interventions.

Although significant improvements occurred in most domains of health, the interventions were ineffective at improving and sustaining physical health measures long-term. Consequently, it can be suggested that synchronous cross-discipline collaboration is successful at improving both psychological and functional outcomes, aspects of well-being historically excluded by disease-centric models of primary care. Furthermore, implementing interventions that include MDT meetings can be cost-effective, as well as contributing to significant improvements made in relation to provider behaviour in the form of medication adjustments. Such medication adjustments are vital in reducing the risk of inappropriate prescriptions, subsequently alleviating the adverse consequences associated with polypharmacy [31].

### Strengths and limitations

To our knowledge, this is the first systematic review conducted on the efficacy of MDT meetings in primary care for individuals living with multimorbidity. This research addresses the prominent gap in knowledge around the care of people with multimorbidity, adding valuable insights into the impact of employing MDT-based care to improve service delivery. This review also expands on previous work by Smith *et al* and Serafino *et al*, achieving a granular analysis by focusing on one aspect of integrated care provision in a primary care setting [16,17]. In addition, this review reaffirms the conclusions obtained by prior literature, that the effectiveness is dependent on the outcome investigated.

Despite a comprehensive search, only four RCTs were identified for inclusion, likely due to strict eligibility criteria. This includes the sole inclusion of the RCTs and the exclusion of qualitative studies. Studies which investigated the efficacy of interventions focused on care coordination and those set in secondary care settings with the involvement of a general physician were also omitted, which restricted the search further. Previous research has highlighted the difficulty in performing RCTs in primary care settings, citing that barriers include challenges with integrating the intervention into usual service delivery [32]. Although multidisciplinary in nature, these studies did not specify synchronous collaboration in primary care. Other potential limitations include that all trials took place within a short timeframe of 12 years, with only one referring to targeting low-income patients. Also, due to clinical heterogeneity, a meta-analysis, a method regarded as gold-standard when synthesising evidence, could not be performed. Nonetheless, a successful narrative synthesis of the outcomes was conducted.

There is debate around the consensus of terminology to describe healthcare teams. Although this review used the terminology most frequently employed by experts, this jargon varies across culture and language. For example, in the UK, ‘MDT’ is most regularly used. Yet, in the USA, a ‘primary care team’ was often described. Although this language fluctuation was incorporated into the search strategy, the only trials eligible for inclusion were from the USA. This could be considered a significant limitation to this review. In addition, further databases could have been incorporated to ensure an exhaustive search. Furthermore, the improvements observed throughout our review cannot be attributed to the MDT meeting alone. The MDTs were just an element within the broader intervention; therefore, is difficult to distil their specific weight to the resulted outcome. This is a clear limitation of the evidence collated, as causality could not be assessed.

### Implications for clinical practice and future research

Additional evidence is urgently required to guide the imminent transition of NHS primary care from delivering fragmented services to an integrated care approach. Most trials in this review focused on improving the delivery of care for older patients living with specific combinations of comorbidities. More trials evaluating interventions targeting multimorbidity more generally are required to inform consensus concerning how to effectively manage this patient population. Furthermore, by neglecting adults of all ages in generating evidence, there will be a perpetuation of the cycle where there is no evidence-base for these individuals, resulting in inappropriate care and increased burden. We therefore recommend that future research should incorporate participants of all ages.

The characteristics of MDT meetings should also be explored further. For example, patient presence was not described by any the trials included. Although patient involvement is not mandatory, coproduction is a recommended feature of integrated care and should be incorporated into future trials. Moreover, the poor reporting of these characteristics has been highlighted, in the hope that this review will encourage researchers to prioritise presenting this information adequately. Decision-makers could then comprehensively assess and implement effective components, to support wider implementation. To achieve this, the ‘Standards of Quality Improvement Reporting Excellence’ and associated practical methods in the interventions could be utilised to ensure adequate reporting of intervention components, to make sure that each component can be replicated.

Further trials located outside of the USA are also required. Although international health policy for managing individuals with multimorbidity is informed by USA approaches to quality improvement and service redesign, the structure of the health system and primary care is vastly different in comparison to other countries. Contributions from other disciplines are more frequent, offering greater opportunities for interprofessional collaboration. The interventions detailed in this review may therefore be unsuitable for a global context and would compromise quality of care. Future trials should appreciate the importance of contextual adaptation, to support the wider implementation of MDTs in primary care.

Barriers to MDT meeting implementation may include assumptions about professional hierarchy, confidence in collaboration, availability of staff and time constraints [33]. MDT meetings may be too complex to integrate successfully into primary care and may cause a substantial increase in a physician workload, especially in rural and other isolated areas which are not well served by healthcare providers. Innovative alternatives to traditional MDT meetings are urgently required to mitigate this burden. The COVID-19 pandemic has caused the rapid adoption of digital technology in healthcare. Recently, significant progress has consequently been achieved regarding the use of technological approaches to improve the provision of primary care and healthcare professionals have become adept at utilising online platforms [34]. Hosting MDT discussions online could promote more efficient ways of collaboration. Pariser *et al* evaluated the feasibility of a telemedicine-hosted, team meeting and concluded that the model meets the needs of both patients living with multimorbidity and their physicians [35]. This mode of delivery would provide an easily accessible and cost-effective alternative to in-person discussions, with no limitations regarding room capacity or location. Policy makers and healthcare professionals should not lose momentum and return to prior inefficient ways of working, when there are clear advantages to communication facilitated by technology. Furthermore, as team varies with size and type of practice, interprofessional care may provide a universal solution to relieving the burden of implementation in primary care. An interprofessional ‘Teamlet Model of Primary Care’ could therefore be a contextually adaptable and sustainable alternative to accommodating MDTs in primary care [36]. However, further research is required regarding dynamics surrounding team-based care, within smaller primary care settings.

## Conclusion

Traditional disease-centric models of multimorbidity management are ineffective, inappropriate and can result in over- and ineffective treatment and fragmented care. Although MDT collaboration is highlighted as a key strategy for delivering comprehensive integrated care, there is a lack of evidence concerning the efficacy of MDT meetings in primary care. The complexity of interventions meant that causality cannot be attributed to the MDT meeting alone. The reported quality of the MDTs was also poor. It is unclear if the results presented here are sufficient to support the widespread implementation of MDT meetings in primary care for adults living with multimorbidity in England’s NHS. There is an urgent need generate more evidence and future research should focus on a broader set of participant characteristics, contextual adaptation, and innovation. Decision makers and clinicians should also take advantage of the recent technological progress in healthcare and apply these digital approaches to facilitate MDT working.

## Additional File

The additional file for this article can be found as follows:

10.5334/ijic.6473.s1 Appendix. Appendix A and B.
